# Evidence for increased interferon type I activity in CD8+ T cells in giant cell arteritis patients

**DOI:** 10.3389/fimmu.2023.1197293

**Published:** 2023-06-16

**Authors:** Marieke van Nieuwland, Idil Esen, Rosanne D. Reitsema, Wayel H. Abdulahad, Yannick van Sleen, William F. Jiemy, Maria Sandovici, Elisabeth Brouwer, Lenny van Bon

**Affiliations:** ^1^ Department of Rheumatology and Clinical Immunology, Hospital Group Twente (Ziekenhuisgroep Twente), Almelo, Netherlands; ^2^ Department of Rheumatology and Clinical Immunology, University of Groningen, University Medical Center Groningen, Groningen, Netherlands; ^3^ School of Medical Sciences, Faculty of Medicine and Health, Örebro University, Örebro, Sweden

**Keywords:** interferon, giant cell arteritis, T cell, vasculitis, large vessel vasculitis, innate immunity, adaptive immunity

## Abstract

**Introduction:**

Giant cell arteritis (GCA) is a vasculitis of the medium- and large-sized arteries. Interferon type I (IFN-I) is increasingly recognized as a key player in autoimmune diseases and might be involved in GCA pathogenesis, however evidence is limited. IFN-I activates Janus kinase/signal transducers and activators of transcription (JAK-STAT) pathways, leading to increased expression of interferon stimulated genes. In this study, IFN-I activity in GCA is explored, focusing on CD8+ T cells.

**Methods:**

Expression of phospho-STAT (pSTAT) 1, 3 and 5 was investigated in IFN-α-stimulated peripheral mononuclear cells (PBMCs) gated separately for CD8+ T cells of patients with GCA (n=18), healthy controls (HC, n=15) and infection controls (n=11) by Phosphoflow method combined with fluorescent cell barcoding technique. Furthermore, IFN-I induced myxovirus-resistance protein A (MxA) and CD8+ T cell expression was investigated by immunohistochemistry in temporal artery biopsies (TAB) of GCA patients (n=20) and mimics (n=20), and in aorta tissue of GCA (n=8) and atherosclerosis patients (n=14).

**Results:**

pSTAT1 expression was increased in IFN-α stimulated CD8+ T cells from GCA patients, whereas no difference was observed in pSTAT3 and pSTAT5 expression. MxA was present in TABs of 13/20 GCA patients compared to 2/20 mimics and in 8/8 GCA+ compared to 13/14 GCA- aorta tissues. MxA location partially co-localized with CD8+T cells.

**Conclusions:**

Our results provide evidence for increased IFN-I activity in CD8+ T cells of GCA patients, both systemically and locally. These findings warrant further investigation regarding IFN-I induced biomarkers and IFN-I related novel therapeutic options in GCA.

## 1 Introduction

Giant cell arteritis (GCA) is the most frequent form of vasculitis of the medium- and large-sized vessels that affects people over 50 years old (1). Two subtypes can be distinguished, cranial GCA (C-GCA) and extracranial large vessel GCA (LV-GCA) (2), which often overlap. Typical C-GCA symptoms include headaches, jaw claudication and blindness, however in LV-GCA nonspecific symptoms such as fever, fatigue and weight loss are common (2). Early GCA diagnosis is key to prevent development of severe complications (3). Nevertheless, this is challenging considering that no single clinical or laboratory test can yet confirm or rule out the diagnosis GCA (4).

Immunopathology of GCA has yet to be fully unravelled and the trigger for the inflammatory response in GCA is still unknown (5, 6). However, it has been previously suggested that viral infections can play a role in initiating the disease, similar to other rheumatic autoimmune conditions (7–10). Interferon type I (IFN-I) is a cytokine that acts as an important defense mechanism in response to viral infections. IFN-I stimulates the Janus kinase/signal transducers and activators of transcription (JAK/STAT) pathways. STAT is activated by phosphorylation (pSTAT), and this activation induces IFN-I stimulated gene (ISG) expression in immune effector cells (11). Elevated expression of ISGs and therefore IFN-I activity has been reported in various autoimmune diseases (12, 13). Recently, IFN-I involvement has been described in patients with anti-neutrophil cytoplasmic antibodies (ANCA)–associated vasculitis and aortitis (14, 15) and a recent small open label study demonstrated that the JAK/STAT inhibitor baricitinib can be effective in GCA (16). Nevertheless, IFN-I activity in GCA has not been studied so far in cells and aorta tissue from GCA patients and it remains unclear whether IFN-I and JAK/STAT signalling play an important role in its pathogenesis.

CD8+ T cells, that can kill infected or damaged cells, are involved in pathogenesis of various autoimmune conditions (17). They produce proinflammatory cytokines in response to self-antigens. Also in GCA pathogenesis, the involvement of CD8+ T cell response is highlighted. Several studies have described presence of CD8+ T cells in inflamed temporal artery biopsies of GCA patients (6, 18, 19). Expansion of CD8+ T cells in response to a viral infection is directly linked to IFN-I signalling (20). Intriguingly, increased expression of a specific interferon stimulated gene (ISG) called interferon induced transmembrane protein 1 (IFITM1) was observed in circulating CD8+ T cells in a limited number of GCA patients by single cell RNA sequencing (18). This suggests involvement of IFN-I signalling in CD8+ T cells in GCA patients.

Unravelling the potential role of IFN-I in GCA can lead to novel diagnostic biomarkers and therapeutic options. To assess responsiveness of CD8+ T cells to IFN-I in GCA, we used Phoshoflow method combined with fluorescent barcoding technique to study several JAK/STAT pathways in circulating CD8+ T cells. In addition, we used immunohistochemistry (IHC) and immunofluorescence to explore IFN-I expression in both C-GCA and LV-GCA patients by investigating IFN-I induced human myxovirus resistance protein 1 (MxA) expression. IFN-I is a cytokine that is not easily measurable in tissue, however expression of MxA can easily be assessed by immunohistochemistry and reliably reflects presence of IFN-I (21).

## 2 Methods

### 2.1 Study population

#### 2.1.1 Peripheral blood

To assess the JAK/STAT pathway and CD8+ T cells in peripheral blood, 18 newly diagnosed treatment naive GCA patients (GCA+), 15 healthy controls (HC) and 11 infection controls (INF; diagnosed with pneumonia or urinary tract infection) from University Medical Center Groningen (UMCG) were used. Patients were diagnosed with GCA based on a positive temporal artery biopsy (TAB) and/or a positive fluorodeoxyglucose-positron emission tomography/computed tomography ([^18^F]FDG-PET/CT) for LV-GCA.

#### 2.1.2 Vascular tissue

To assess IFN-I and CD8+ T cells in inflamed tissue, TAB and aorta tissues of GCA patients were used to include both C-GCA (TAB) and LV-GCA (aorta). TABs of 20 patients diagnosed with GCA (GCA+) and 20 patients suspected of but not diagnosed with GCA (GCA-), based on clinical diagnosis by the treating physician after six months follow-up, were retrospectively selected. TABs were originally performed for diagnostic purposes at Hospital Group Twente. Aorta tissue of 8 GCA+ patients diagnosed based on histology and 14 control patients with atherosclerosis (GCA-) were retrospectively selected from patients who underwent aortic aneurysm surgery in the UMCG.

The experiments were performed in accordance with the declaration of Helsinki and approved by the University Medical Center Groningen review board (METc2012/375 for HC and METC2010/222 for GCA+ patients), or were not subjected to the medical research involving human subjects act. Informed consent was obtained from prospectively included patients.

### 2.2 Fluorescent cell barcoding

Frozen PBMCs were thawed and re-suspended to 5x10^6^ cells/ml in RPMI1640 (Cambrex Bio Science, Verviers, Belgium) supplemented with 10% FCS and 50 mg/ml gentamycin (Gibco, Scotland, UK). The cell suspension was aliquoted into polypropylene tubes (5x10^5^ cells per tube), labeled with BUV395-anti-CD19 (BD), and stimulated with IFN-α. Next, samples were incubated for 30 minutes at 37^0^C and 5% CO_2_. After stimulation, cells were fixed with Cytofix (BD) and Phosphoflow protocol was used for measuring phosphorylated STAT1/3/5/. The samples were stained with AF647-anti-phospho-STAT1 (pSTAT1) (BD), PE-anti-pSTAT3 (BD), AF488-anti-pSTAT5 (BD), BUV737-anti-CD3 (BD), Pe-CY7-anti-CD4 (BD), and BV711-anti-CD45RO (BD) antibodies. After staining, the cells were analyzed on FACSymphony (Becton Dickinson). Data were plotted using the Kaluza software package (Beckman Coulter). Within CD8+ T cells, CD4+ T cells, B cells, and monocytes, different simulation tubes were identified based on their fluorescent cell barcoding signature, gated separately, and analyzed as individual samples for the expression of pSTAT1, pSTAT3 and pSTAT5. Unstimulated samples were used for setting the linear gates to delineate positive and negative populations.

### 2.3 Immunohistochemistry

To assess the presence of IFN-I induced MxA and CD8+ T cells in inflammatory tissue, immunohistochemistry (IHC) on both TAB and aorta tissue of GCA+ and GCA- patients was performed. Furthermore, presence of plasmacytoid dendritic cells (pDCs), which are the main IFN-I producers, at the site of inflammation was assessed by staining for CD303 in TABs. Formalin-fixed and paraffin-embedded tissues were deparaffinized. After antigen retrieval, slides were incubated with primary antibodies overnight (MxA, see **Supplementary Table S1**) at 4°C or for 60 minutes at room temperature (CD8 and CD303, see **Supplementary Table S1**). After peroxidase blocking, tissue was incubated with the secondary antibody tagged with horseradish peroxidase (HRP) for 40 minutes at room temperature. The tissues were subsequently incubated with 3‐amino‐9‐ethylcarbazole (DAKO, Glostrup, Denmark) for peroxidase activity detection, and with haematoxylin (MERCK, Kenilworth, NJ, USA) as a counterstain. All slides were scanned using a Nanozoomer Digital Pathology Scanner (NDP Scan U 10074‐01; Hamamatsu Photonics K.K., Shizuoka, Japan). Two authors (MvN and LvB) separately assessed protein expression semi-quantitatively in all vessel layers (i.e. intima, media, adventitia) by using a score ranging from 0-4 (0=none, 4=high number of positive cells (>50%)). The mean score per vessel layer was used.

### 2.4 Immunofluorescence

To show colocalization of MxA and CD8+, double-labelling of MxA and CD8+ was performed on three TABs and three aorta tissues of GCA+ patients. Sequential opal immunofluorescence staining was performed to confirm the co-expression of MxA and CD8. Formalin-fixed, paraffin-embedded TAB and aorta sections were deparaffinized and rehydrated, followed by 15 minutes of antigen retrieval with Tris-EDTA buffer (pH9) in a microwave. Tissues were incubated with primary antibodies targeting MxA (see **Supplementary Table S2**) overnight at 4˚C. Sections were then blocked for endogenous peroxidase activity and incubated with secondary antibodies tagged with horseradish (HRP) peroxidase. Opal 620 fluorophore (Akoya Biosciences, Delaware) was developed by incubation for 10 minutes. Bound antibodies and unspecific fluorophores were stripped by 15 minutes of antigen retrieval with Tris-EDTA buffer (pH9) in a microwave. Tissues were again incubated with primary antibodies targeting CD8 (see **Supplementary Table S2**) for 1 hour at RT. Sections were then incubated with secondary antibodies tagged with horseradish (HRP) peroxidase. Opal 650 fluorophore (Akoya Biosciences, Delaware) was developed by incubation for 10 minutes. Bound antibodies and unspecific fluorophore were stripped by 5 minutes of antigen retrieval with Tris-EDTA buffer (pH9) in a microwave. Sections were then incubated with DAPI as counterstain and sealed. Image cubes were captured at a magnification of 20× and 40x using Nuance Multispectral Imaging System 3.0.1 (PerkinElmer, Waltham, MA, USA) using NuanceFX 3.0.1 software (PerkinElmer). Spectral unmixing was performed with spectral libraries of each fluorophore assigned different colours, subtracting the background autofluorescence.

### 2.5 Statistical analysis

All graphs were generated using GraphPad Prism version 5 (GraphPad Software Inc., La jolla, CA, USA). To assess differences between pSTAT1 expression, a Kruskal Wallis test and separate Mann Whitney U tests were performed. Generally, P values < 0.05 were considered statistically significant.

## 3 Results

### 3.1 pSTAT1 expression is increased in circulating CD8+ T cells after IFN-α stimulation in GCA+ patients

**Figure 1:**
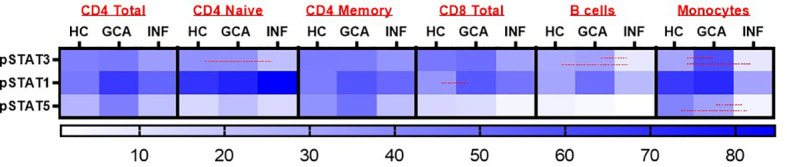
Heatmap representation of pSTAT3, pSTAT1 and pSTAT5 response after IFN-α stimulation in CD4+ Total, CD4 Naïve, CD4 Memory, CD8+ Total, B cells and monocytes. Red lines were used to show statistically significant differences between groups (p<0.05). In CD8+ T cells, a significant difference between GCA+ patients and HCs was observed in pSTAT1 expression. HC, healthy controls; GCA, treatment naïve GCA+ patients; INF, infection controls.

To investigate responsiveness of circulating PBMCs to IFN-α stimulation, we performed fluorescent cell barcoding. **Supplementary Table S3** shows patient characteristics of treatment-naïve GCA patients (n=18), age-matched HCs (n=15) and INF (n=11) used for barcoding experiments. 16 GCA patients met the 2022 ACR/EULAR GCA classification criteria. Expression of pSTAT1, pSTAT3 and pSTAT5 was investigated. Interestingly, pSTAT1 expression was increased in CD8+ T cells of GCA+ patients compared to HC, and this difference was statistically significant (P<0.05). Stratification for memory and naive T cells showed that pSTAT1 was specifically upregulated in memory CD8+ T cells (**Supplementary Figure S1**). After IFN-α stimulation, pSTAT1, pSTAT3 and pSTAT5 were expressed in all assessed cell types, however no difference between GCA+ patients and HC was observed in CD4+ total, CD4+ naive and CD4+ memory T cells, B cells and monocytes. In B cells and monocytes, pSTAT3 was upregulated and in monocytes, pSTAT3 and pSTAT5 were upregulated in GCA patients compared to INF, but not compared to HC (p<0.05) (**Figure 1**). Body Mass Index and HbA1c did not correlate to pSTAT1 expression in GCA+ patients and HCs.

### 3.2 IFN-I induced MxA is detected in GCA affected TABs and aorta tissue

**Figure 2:**
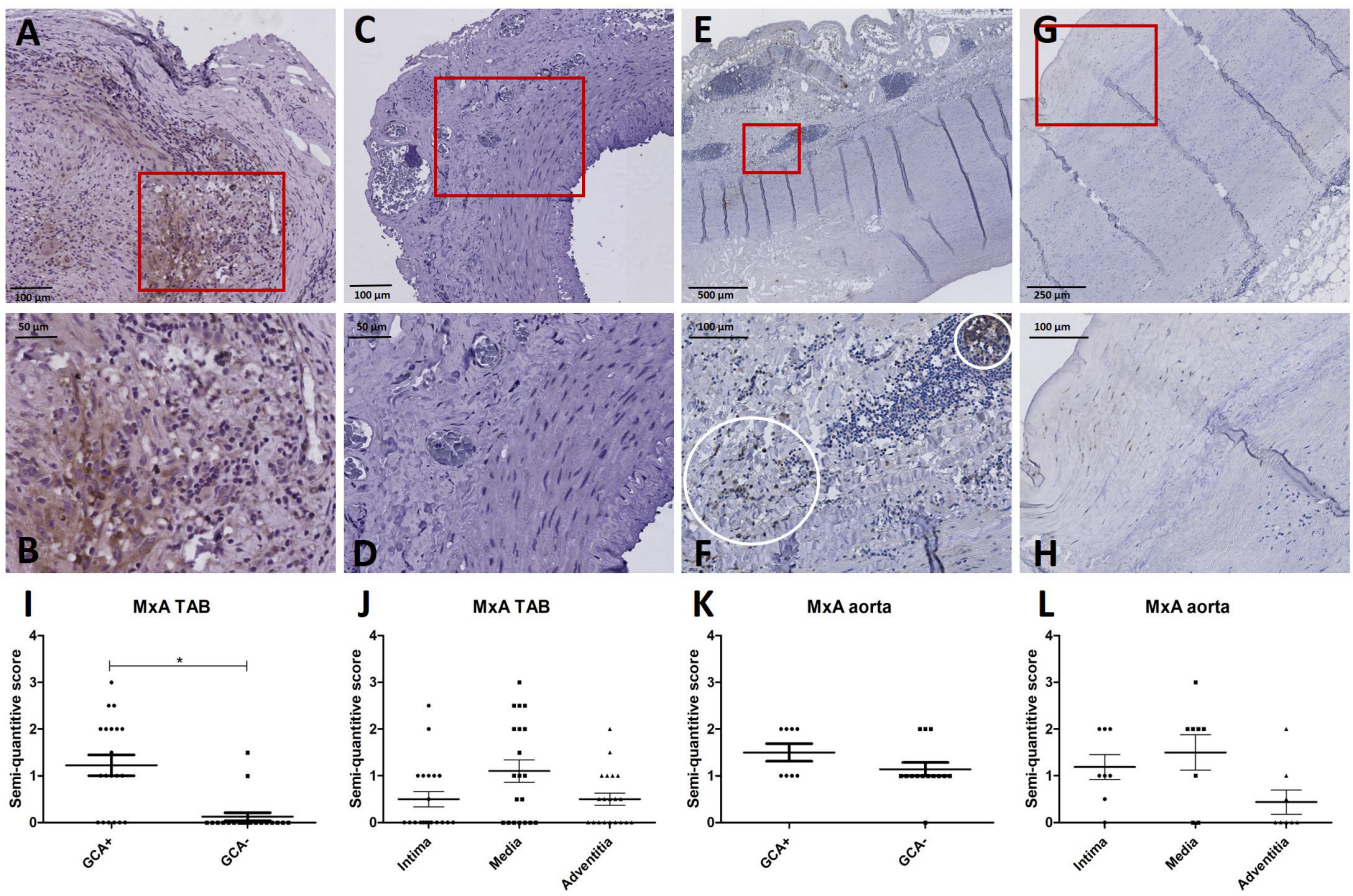
MxA immunohistochemistry (brown) performed on temporal artery biopsies (TAB) and aorta tissues of giant cell arteritis (GCA)+ and GCA- patients. Representative MxA and enlarged area in TAB of a GCA+ patient **(A, B)** and a GCA- patient **(C, D)**; representative MxA and enlarged area in aorta tissue of a GCA+ patient **(E, F)** and a GCA- patient **(G, H)**; semi-quantitative scoring of MxA expression in TAB for GCA+ and GCA- patients **(I)** and for different vessel layers in GCA+ patients **(J)**; semi-quantitative scoring of MxA expression in aorta tissue for GCA+ and GCA- patients **(K)** and for different vessel layers in GCA+ patients **(L)**.

**Supplementary Table S4** shows patient characteristics of the retrospective cohort used for IHC of TABs in GCA+ (n=20) and GCA- (n=20) patients. All GCA+ patients met the 2022 ACR/EULAR GCA classification criteria. To assess the cellular response to IFN-I at the site of inflammation, MxA expression was assessed. MxA was detected in GCA affected vessel walls of 14/20 GCA+ patients with biopsy-proven C-GCA. MxA was only detected in TABs of 2/20 GCA- patients. The difference between GCA+ and GCA- patients was statistically significant (P<0.05). **Supplementary Table S4** also shows patient characteristics of aorta tissue IHC LV-GCA patients. MxA was expressed in 8/8 GCA+ patients and in 13/14 GCA- patients with atherosclerosis. In TABs and aorta tissue, MxA was expressed in all vessel layers varying between patients (**Figure 2**). As pDCs are the main producers of IFN-I, CD303 expression was assessed in TABs of GCA+ and GCA- patients. No pDCs were detected in both GCA+ and GCA- TABs, suggesting an alternative cellular source for IFN-I production (**Supplementary Figure S2**).

### 3.3 CD8+ T cells are detected in multiple vessel wall layers in GCA affected TABs while mainly present in the adventitia of GCA affected aorta tissue

**Figure 3:**
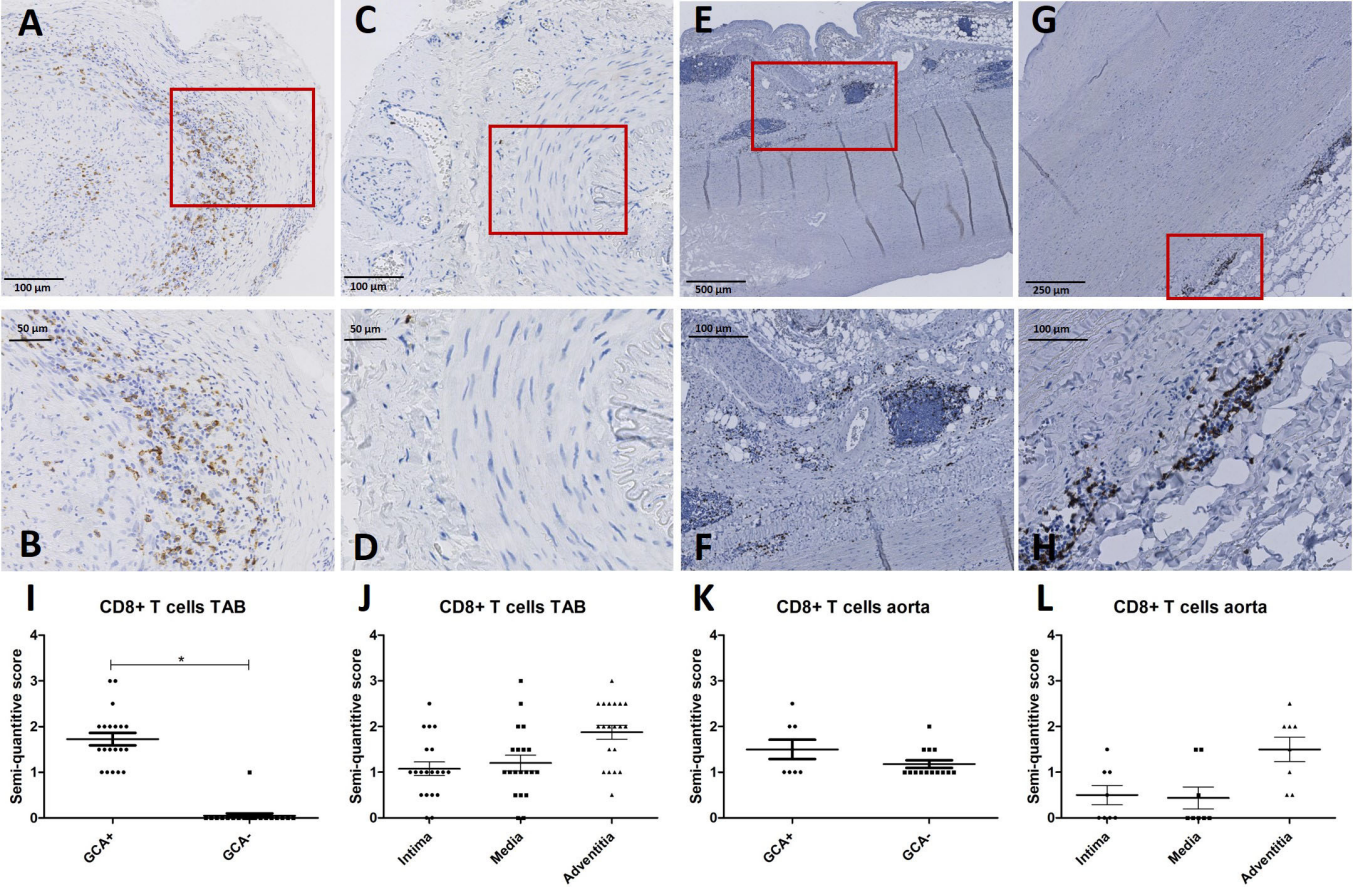
CD8+ immunohistochemistry (brown) performed on temporal artery biopsies (TAB) and aorta tissues of giant cell arteritis (GCA)+ and GCA- patients. Representative CD8+ and enlarged area in TAB of a GCA+ patient **(A, B)** and a GCA- patient **(C, D)**; representative CD8+ and enlarged area in aorta tissue of a GCA+ patient **(E, F)** and a GCA- patient **(G, H)**; semi-quantitative scoring of CD8+ expression in TAB for GCA+ and GCA- patients **(I)** and for different vessel layers in GCA+ patients **(J)**; semi-quantitative scoring of CD8+ expression in aorta tissue for GCA+ and GCA- patients **(K)** and for different vessel layers in GCA+ patients **(L)**. Biopsy tissue used in A-D are from the same patients as in **Figure 2**.

Considering pSTAT1 upregulation observed in in circulating CD8+ T cells of GCA+ patients in our fluorescent cell barcoding data, we subsequently assessed the presence of CD8+ T cells in the same TABs and aorta tissue of GCA+ and GCA- patients as used for MxA stainings. CD8+ T cells were observed in TABs of 20/20 GCA+ patients and 1/20 GCA- patients. In aorta tissue, CD8+ was expressed in 8/8 GCA patients and 14/14 GCA- patients with atherosclerosis. CD8+ was expressed in all vessel layers of TABs and was mainly expressed in the adventitia of aorta tissue (**Figure 3**).

### 3.4 MxA and CD8+ partially colocalize in TAB and aorta tissue of GCA patients

**Figure 4:**
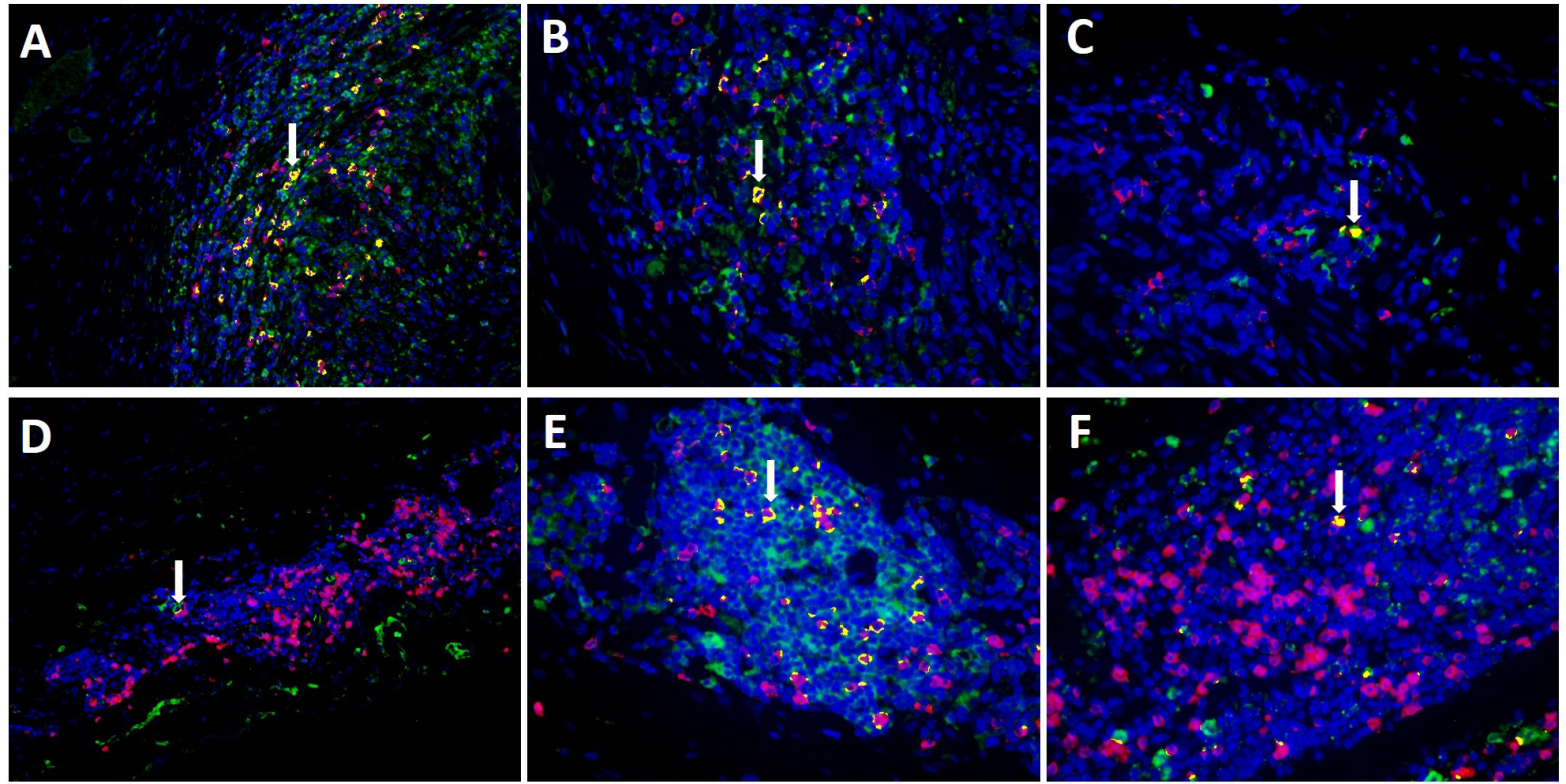
Co-expression (yellow) of MxA (green) and CD8+ T cells (pink) in TAB of 3 C-GCA patients **(A–C)** and aorta tissue of 3 LV-GCA patients **(D–F)** at 20x **(A, D)** and 40x **(B, C, E, F)** magnification. Nuclei were stained with DAPI (blue).

Evaluating MxA and CD8+ immunohistochemistry, expression of both was observed in similar cell areas. To confirm this observation, double-labelling of MxA and CD8+ was performed using immunofluorescence. **Figure 4** shows double-labelling results in TABs and aorta tissue of GCA+ patients (see also **Supplementary Figure S3**). Interestingly, colocalization of MxA and CD8+ was more prevalent in TABs compared to aorta tissue and colocalization patterns in TABs varied for each patient. Unsurprisingly, MxA was not only expressed by CD8+ T cells, but other cell types in the vessel wall also expressed MxA (**Figure 4**).

## 4 Discussion

In the present study we show that peripheral blood CD8+ T cells of GCA patients have upregulated pSTAT1 expression compared to HCs after IFN-α stimulation. In addition, we show that IFN-I induced MxA is present in TAB and aorta tissue of GCA patients. Furthermore, we document expression of MxA by the CD8+ T cells mainly in TABs of patients with GCA. This evidence altogether suggests increased IFN-I activity in GCA patients, possibly linked to CD8+ T cells.

It has been suggested that infections or other IFN-I mediated inflammatory triggers might activate the innate and adaptive immune system and DCs in the vessel wall (8, 22). As suggested by the pathogenesis theory of GCA which implicates activation of resident vascular dendritic cells possibly by a viral trigger, IFN-I might play a role in immunopathology of GCA. Research in other systemic auto-immune diseases such as systemic lupus erythematosus, Sjögren’s syndrome and rheumatoid arthritis showed increased levels of IFN-I. Furthermore, IFN response gene expression was described to decline after prednisolone treatment in GCA, implying it was present in disease initiation (23). Using single cell RNA sequencing, we have previously shown that IFITM1 was overexpressed in circulating CD8+ T cells in GCA patients (18). Furthermore, it has been described that phosphorylation of STAT1 and STAT2 leads to a higher expression of ISGs *via* the JAK/STAT pathway (24), and STAT1 and STAT2 target genes are present in tissue transcriptome of GCA affected arteries (25). As CD8+ memory T cells in particular responded with increased phosphorylation of STAT1 after IFN-α stimulation when comparing to HC in our study, this further supports the importance of the IFN-I pathway in GCA.

Our results on MxA expression in tissue are in line with previously described MxA expression in TABs of 4 patients (26), as we observe MxA expression in the majority of GCA+ patients in both TAB and aorta tissue. In search for the answer if IFN-I is produced locally in the vessel wall we investigated the presence of pDCs, the most potent producers of IFN-I (27). In TAB vessels walls pDCs were not present. Further research should elucidate the source of IFN-I (systemic or local by cells other than pDCs). As previously described, memory CD8+ T cells predominantly reside in GCA affected vessel walls (19). Although CD8+ T cells were present in both TAB and aorta tissue, they only partially colocalized with MxA as shown by our immunofluorescence double stainings. This can be explained by multiple cell types responding to IFN-I stimulation, which was confirmed by the fact that CD4+ total, CD4+ naive and CD4+ memory T cells, CD8+ T cells, B cells and monocytes all expressed pSTAT1, pSTAT3 and pSTAT5 after IFN-α stimulation. Based on MxA expression, other important cell types such as macrophages or B-cells in tissue might also colocalize with MxA (28, 29).

A major strength of this study was that we investigated both C-GCA and LV-GCA tissue as well as circulating PBMCs. By stimulating cells with IFN-α to look at pSTAT1 expression and assessment of MxA expression in GCA affected tissue, different aspects of IFN-I responsiveness were investigated. In addition, appropriate control groups were included for both peripheral blood and vascular tissue. Limitations of this study include the use of different patient populations for peripheral blood and vascular tissue studies. Additionally, lipid levels were unavailable in our peripheral blood cohort dataset, possibly introducing bias since atherosclerosis is associated with systemic levels of IFN-I (30, 31). In aorta tissues, MxA was also expressed in atherosclerosis patients, which is confirmed in previous literature (30). Furthermore, inevitably for GCA, many patients were already treated with glucocorticoids at time of TAB, possibly influencing MxA expression in IHC TAB stainings. Also, experiments in circulating PBMCs were performed using new, treatment naive GCA patients, and aorta tissues used for IHC experiments were probably from later disease stages compared to TABs. Future research is needed to substantiate the evidence provided in this study. It would be useful to study GCA disease progression as well as comparison to patients with polymyalgia rheumatica to obtain more insight in IFN-I mechanisms in GCA patients. Furthermore, CD8+ T cell subsets should be studied to further specify their role in IFN-I activity. Nevertheless, as our results all indicate a possible role for IFN-I in GCA immunopathology more research is needed to further elucidate IFN-I responsiveness in different GCA subtypes.

In conclusion, we report the expression of MxA in GCA patients, which may reflect increased IFN-I activity., Also, we show increased pSTAT1 in circulating CD8+ T cells of GCA patients, providing evidence for IFN-I activity both systemically and locally. Therefore, our data support a role for IFN-I in GCA immunopathogenesis, possibly associated with cellular senescence. Interestingly, MxA was not detected in all GCA+ patients and therefore, IFN-I assessment in GCA+ patients might add to stratification and personalized treatment in the future.

## Data availability statement

The raw data supporting the conclusions of this article will be made available by the authors, without undue reservation.

## Ethics statement

The studies involving human participants were reviewed and approved by METc2012/375 and METC2010/222 University Medical Center Groningen review board. The patients/participants provided their written informed consent to participate in this study.

## Author contributions

All authors listed have made a substantial, direct, and intellectual contribution to the work and approved it for publication.

## Supplementary material

Supplementary Figure 1 pSTAT1 positivity (%) after IFN-α stimulation in CD8+ T cells of HC, INF and GCA patients stratified for CD8 memory and CD8 naïve T cells. A statistically significant difference between GCA+ patients and HCs was observed in pSTAT1 expression of CD8 memory T cells.

Supplementary Figure 2 Representative CD303 staining in TAB of a GCA+ patient **(A)**, a GCA- patient **(B)** and thymus tissue as a positive control **(C)**. No pDCs were detected in both GCA+ and GCA- patients. Red squares indicate CD303 positive cells.

Supplementary Figure 3 Negative control staining for the co-expression of MxA and CD8+ T cells.
