# Supplementary material for: Evidence for increased interferon type I activity in CD8+ T cells in giant cell arteritis patients
Source: Front Immunol. 2023 Jun 16;14:1197293. doi: 10.3389/fimmu.2023.1197293 (PMC10312374; doi:10.3389/fimmu.2023.1197293)
Supplement: Supplementary file 1 [file Table_1.docx]

**Supplementary Table S1** Antibodies for immunohistochemistry

| **Target** | **Antigen retrieval** | **Manufacturer & code** | **Host** | **Isotype** | **Dilution** | **Secondary antibody** | **Manufacturer & Code** | **Dilution** |
| --- | --- | --- | --- | --- | --- | --- | --- | --- |
| **MxA** | pH9 | R&D systems, AF7946 | Goat | IgG | 0.3 µg/mL | Anti-goat IgG-HRP | DAKO, P0449 | 1:50 |
| **CD8** | pH9 | Abcam, ab75129 | Mouse | IgG1 | prediluted | Envision anti-mouse HRP | DAKO, K4006 | Prediluted |
| **CD303** | PH9 | Dendritcs, DDX0043 | Mouse | IgG1 | 1:100 | n/a* |  |  |

*****Performed on Ventana Benchmark Ultra stainer

**Supplementary Table S2** Antibodies for multiplex immunofluorescence

| **Target** | **Antigen retrieval** | **Manufacturer & code** | **Host** | **Isotype** | **Dilution** | **Secondary antibody** | **Manufacturer & Code** | **Dilution** |
| --- | --- | --- | --- | --- | --- | --- | --- | --- |
| **MxA** | pH9 | R&D systems, AF7946 | Goat | IgG | 0.3 µg/mL | VisUCyte anti-goat IgG-HRP | R&D systems, VC004 | Prediluted |
| **CD8** | pH9 | Abcam, ab75129 | Mouse | IgG1 | prediluted | Envision anti-mouse HRP | DAKO, K4006 | Prediluted |

| Supplementary Table S3 patient characteristics fluorescent barcoding | | | |
| --- | --- | --- | --- |
|  | **HCs (n=15)** | **GCA+ (n=18)** | **INF (n=11)** |
| Age, years, mean (SD) | 67 (9.5) | 68 (9.6) | 78 (12.1) |
| Female, *n* (%) | 10 (67) | 11 (61) | 3 (27) |
| Body Mass Index in kg/m^2^; median (IQR) | 25.6 (23.4-28.4) | 22.3 (21.5-26.1) | n/a |
| HbA1C in mmol/mol; median (IQR) | 39 (37-42) | 43 (39-44.8) | n/a |
| ESR in mm/hr; median (IQR) | 6.0 (3.0-8.75) | 73.5 (49.5-99.0) | 98.0 (72.0-109.0) |
| CRP in mg/L; median (IQR) | 2.0 (0.6-5.0) | 41.0 (23.8-90.5) | 76.0 (61.0-109.0) |

HC = healthy controls; GCA+ = patients with giant cell arteritis; IFN = infection controls; ESR = erythrocyte sedimentation rate; CRP = C-reactive protein. No glucocorticoid treatment was started in all groups.

**Supplementary Table S4** patient characteristics immunohistochemistry

|  | **TAB ZGT cohort** | | **Aorta tissue UMCG cohort** | |
| --- | --- | --- | --- | --- |
|  | GCA+^1^ (n=20) | GCA-^2^ (n=20) | GCA+^3^ (n=8) | GCA-^4^(n=14) |
| Biopsies performed; year(s) | 2017-2018 | 2017-2019 | 2019 | 2019 |
| Age in years; mean (SD) | 73.1 (8.9) | 73.5 (10.3) | 70.8 (8.1) | 68.6 (7.1) |
| Female; n (%) | 16 (80.0) | 12 (60.0) | 4.0 (50.0) | 9.0 (75.0) |
| ESR in mm/hr; median (IQR) | 89.0 (47.0-101.0) | 38.50 (10.3-60.3) | 15.0 (7.0-30.5) | 15.0 (3.5-22.0) |
| CRP in mg/L; median (IQR) | 49.5 (31.0-107.3) | 10.0 (1.0-66.5) | 7.5 (5.0-21.3) | 7.0 (5.0-17.5) |
| Prednisone; n(%)  ≥3 days at time of biopsy; |  |  |  |  |
| <10 mg/day  10-20 mg/day  20-40 mg/day  >40 mg/day  1000 IV mg/day* | 1 (5.0) 0 (0.0) 6 (30.0) 10 (50.0) 1 (5.0) | 0 (0.0) 2 (10.0)  4 (20.0)  4 (20.0)  0 (0.0) |  |  |

TAB = temporal artery biopsy; ZGT = Hospital Group Twente; UMCG = University Medical Center Groningen; ESR = erythrocyte sedimentation rate; CRP = C-reactive protein; ^1^patients with GCA based on clinical diagnosis after six months follow-up; ^2^patients suspected of who were not diagnosed with GCA based on clinical diagnosis after six months follow-up; ^3^ *Intravenous methylprednisolone started ≥ 3 days before biopsy. No LV-GCA patients were treated with prednisone at time of aorta biopsy.
