# Supplementary figures and images for: Evidence for increased interferon type I activity in CD8+ T cells in giant cell arteritis patients
Source: Front Immunol. 2023 Jun 16;14:1197293. doi: 10.3389/fimmu.2023.1197293 (PMC10312374; doi:10.3389/fimmu.2023.1197293)

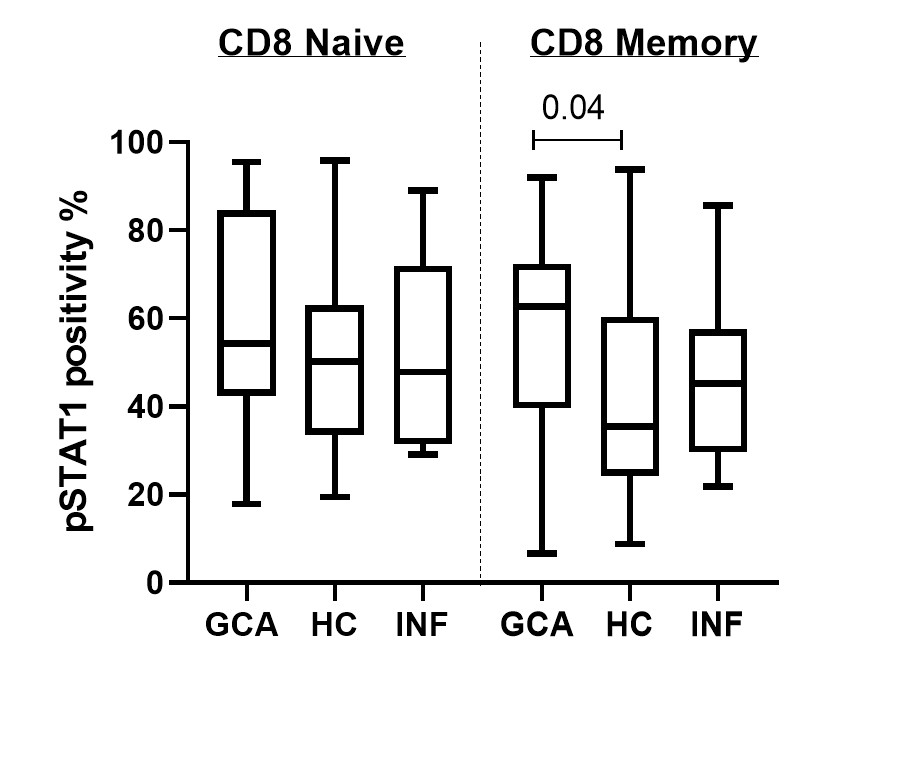

Supplement: Supplementary Figure 1 — pSTAT1 positivity (%) after IFN-α stimulation in CD8+ T cells of HC, INF and GCA patients stratified for CD8 memory and CD8 naïve T cells. A statistically significant difference between GCA+ patients and HCs was observed in pSTAT1 expression of CD8 memory T cells. [file Image_1.jpeg]

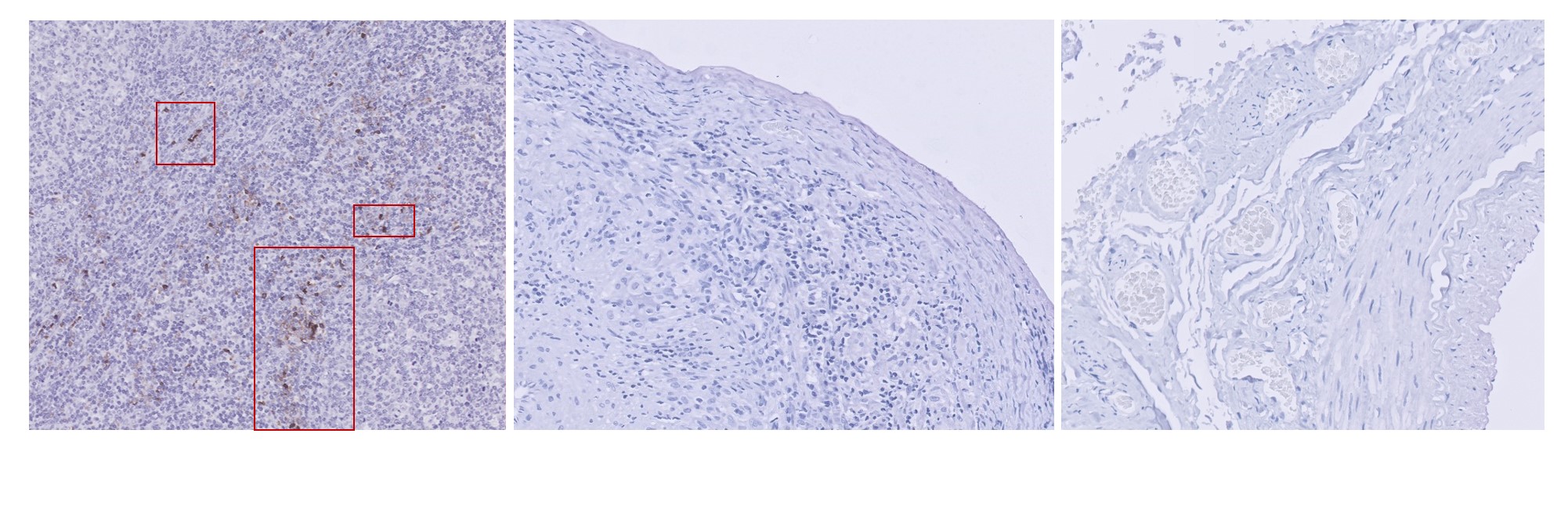

Supplement: Supplementary Figure 2 — Representative CD303 staining in TAB of a GCA+ patient (A), a GCA- patient (B) and thymus tissue as a positive control (C). No pDCs were detected in both GCA+ and GCA- patients. Red squares indicate CD303 positive cells. [file Image_2.jpg]

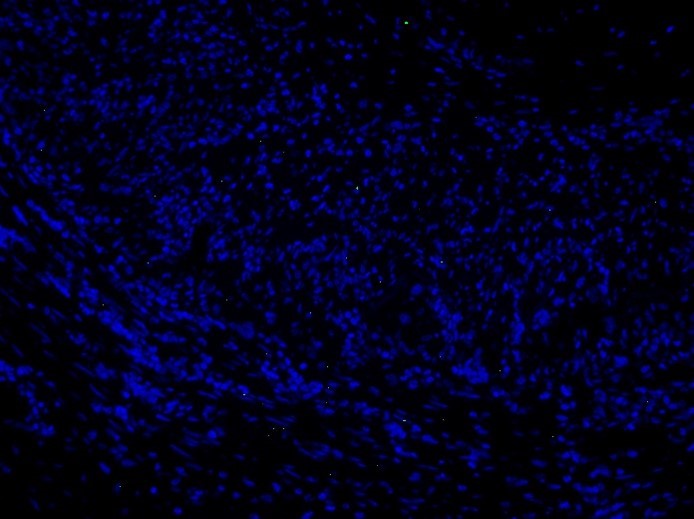

Supplement: Supplementary Figure 3 — Negative control staining for the co-expression of MxA and CD8+ T cells. [file Image_3.jpeg]
